# An Unusual Case of Asystole Occurring during Deep Brain Stimulation Surgery

**DOI:** 10.1155/2016/8930296

**Published:** 2016-04-27

**Authors:** Ha Son Nguyen, Harvey Woehlck, Peter Pahapill

**Affiliations:** ^1^Neurosurgery, Medical College of Wisconsin, Milwaukee, WI 53226, USA; ^2^Anesthesiology, Medical College of Wisconsin, Milwaukee, WI 53226, USA; ^3^Neurosurgery, Clement J. Zablocki VA Medical Center, Milwaukee, WI 53295, USA

## Abstract

*Background*. Symptomatic bradycardia and hypotension in neurosurgery can produce severe consequences if not managed appropriately. The literature is scarce regarding its occurrence during deep brain stimulation (DBS) surgery.* Case Presentation*. A 67-year-old female presented for left DBS lead placement for essential tremors. During lead implantation, heart rate and blood pressure dropped rapidly; the patient became unresponsive and asystolic. Chest compressions were initiated and epinephrine was given. Within 30 seconds, the patient became hemodynamically stable and conscious. A head CT demonstrated no acute findings. After deliberation, a decision was made to complete the procedure. Assuming the etiology of the episode was the Bezold-Jarisch reflex (BJR), appropriate accommodations were made. The procedure was completed uneventfully.* Conclusion*. The episode was consistent with a manifestation of the BJR. The patient had a history of neurocardiogenic syncope and a relatively low-volume state, factors prone to the BJR. Overall, lead implantation can still occur safely if preventive measures are employed.

## 1. Background

Deep brain stimulation (DBS) has become a widely accepted therapy for adjustable, reversible modulation of brain function. Rare, but notable, intraoperative complications include critical hemodynamic instability, particularly symptomatic bradycardia and hypotension, which has been associated with venous air embolism [1]. To the authors' knowledge, there has been no occurrence linked to the Bezold-Jarisch reflex (BJR), a cardioinhibitory reflex that has been associated with states of hypovolemia. Herein, the authors describe a patient who exhibited acute, dramatic asystole during DBS surgery. The episode was consistent with the BJR. After appropriate measures were pursued, the patient underwent an uneventful implantation of her electrode.

## 2. Case Report

### 2.1. Initial Presentation

A 67-year-old right-handed female with a history of neurocardiogenic syncope, irritable bowel syndrome, and hypothyroidism had been suffering from essential tremors for 25 years. Notably, she also had an extensive family history of neurocardiogenic syncope. Exam revealed bilateral upper extremity grade 2/4 intention tremors. Baseline vital signs included height 167 cm, weight 46 kg, blood pressure 90–100s/50–60s, and heart rate 60–90s.

### 2.2. Initial DBS Implantation

**Figure 1:**
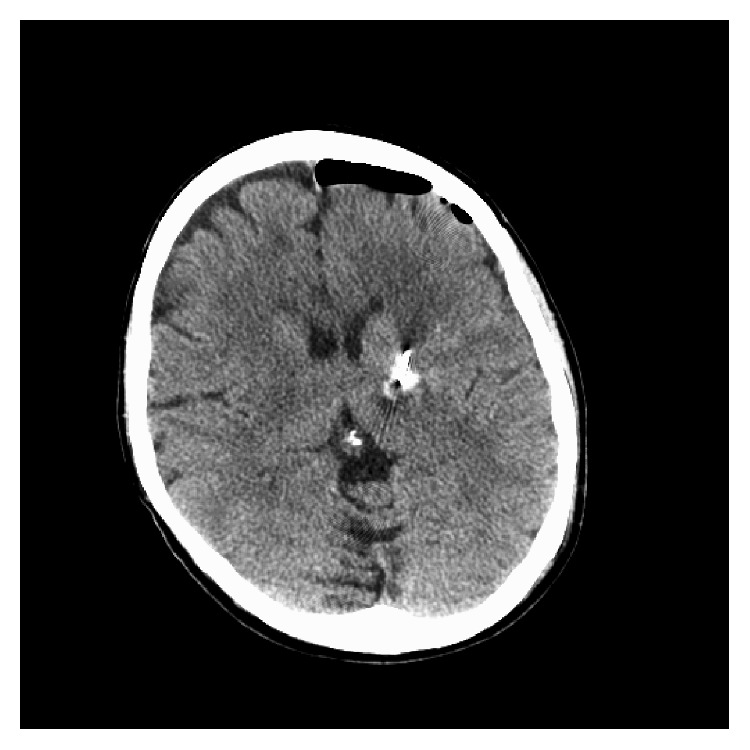
CT head demonstrated a small hemorrhage at the tip of the lead.

**Figure 2:**
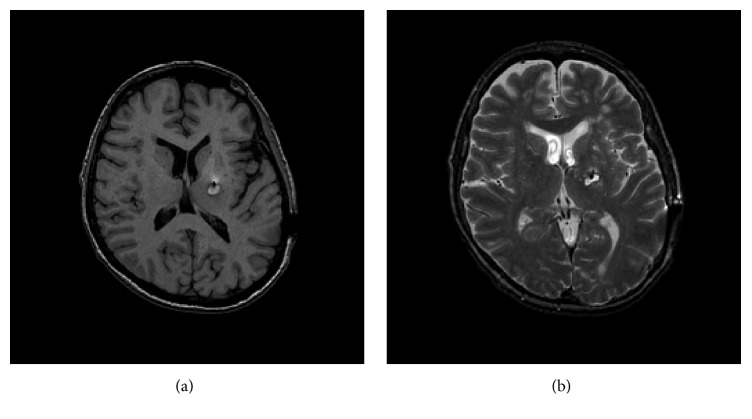
(a) An MRI brain confirmed a small resolving hematoma at the tip of the lead. (b) An MRI brain confirmed a small resolving hematoma at the tip of the lead.

She underwent a Leksell frame-based placement of a left VIM DBS with microrecording, resulting in a significant lesion effect and intraoperative tremor arrest with macrostimulation. Immediately, postoperatively, the patient demonstrated mild difficulty with coordination and speech production, as well as an odd sensation in the right hand. A CT head demonstrated a small hemorrhage at the tip of the lead (Figure 1). At the first programming session, her initial postoperative symptoms, including her lesion effect, had resolved and she demonstrated no benefits from stimulation. An MRI brain confirmed a small resolving hematoma at the tip of the lead (Figures 2(a) and 2(b)); consequently, further programming was held off until the hematoma resolved. A month later, DBS programming again demonstrated no benefits. Revision surgery was recommended.

### 2.3. Initial Revision Attempt

During application of the Leksell stereotactic frame, the patient felt nauseated and promptly became unresponsive. Vital signs were stable. The frame was removed and the patient was placed supinely. Within minutes, she was back to neurologic baseline. A CT head demonstrated no acute findings. The case was cancelled, and the patient was admitted for further evaluation. Work-up included medicine, neurology, psychiatry, and cardiology consultations. No structural heart disease or malignant arrhythmias were identified. Further history revealed several recent fainting episodes. The present episode was attributed to neurocardiogenic syncope. She was also evaluated for a potential psychiatric component given the fact that this occurred a few days away from what would have been her late husband's birthday and a few weeks from the one-year anniversary of his death. A diagnosis of major depressive disorder related to grief was made and a trial of mirtazapine was started. She reported improvement in mood, appetite, and sleep.

### 2.4. Revision Surgery

Three months later, frame placement ensued without issues. The previous burr hole was exposed. After microelectrode mapping, the DBS electrode was placed. Prior to test macrostimulation through the DBS lead, the patient complained of severe nausea. HR decreased quickly from 80–90s to 20–30s and blood pressure from 120s/60s to 60s/30s. The patient became unresponsive and asystolic; respiratory rate remained stable at 10–16. Chest compressions were initiated immediately by the movement disorder neurologist and administration of epinephrine 0.1 mg IV occurred. Subsequently, within 30 seconds, the patient demonstrated return of hemodynamic stability and became responsive, answering simple questions. HR was in the 90s to 100s, and BP was 90s/50s. The procedure was stopped. The DBS electrode and stereotactic arc, but not frame, were removed and the incision was closed. A stat CT head did not demonstrate any abnormality.

Extensive discussions occurred with the patient, the anesthesia team, the neurology team, and the patient's family. The decision was made to bring her immediately back to the operating room and complete the procedure by implanting the DBS electrode as planned. Believing the episode was due to the BJR, the patient was treated with glycopyrrolate and a 20 mL/kg normal saline bolus. There was no recurrence of bradycardia, hypotension, or asystole. Test stimulation through the implanted DBS electrode revealed tremor arrest. There were no postoperative concerns and the patient was discharged the following morning. The patient was seen at 2 weeks' follow-up for adjustment of her DBS settings, which was left at C(+)0(−), 2.2 v, 60 uS, and 150 Hz with tremor arrest and no side effects. At 14 months, she continued to do well and was considering placement of a right DBS electrode to control her remaining left-sided tremors.

## 3. Discussion

We believe the described episode was consistent with the BJR reflex. This reflex is a cardioinhibitory reflex that has been associated with states of hypovolemia [2]. Its afferent limb is facilitated by cardiac receptors via nonmyelinated type C vagal fibers [3]. An initial response to hypovolemia is sensed in the carotid sinus baroreceptors, causing a compensatory phase with increased heart rate, vasoconstriction, and contractility [3]. However, with an empty hypercontractile ventricle, stimulation of the intramyocardial C fibers can potentiate a sudden withdrawal of sympathetic outflow, increasing vagal tone, which can trigger bradycardia and hypotension [3]. The reflex has been described during spinal anesthesia, due to the decrease in preload, and during shoulder arthroscopic surgery interscalene brachial plexus block, due to venous blood pooling associated with the sitting position. A debatable predisposing factor has been the use of exogenous epinephrine (as a local anesthetic), which may prompt beta-adrenergic effects that increase cardiac contractility, triggering reflex arterial vasodilation and bradycardia [4, 5]. Treatment options include immediate fluid resuscitation, vagolytics (atropine and glycopyrrolate) [6, 7], and early use of IV epinephrine [2]. Other reported therapeutic medications include ondansetron [7], metoprolol [8], and ephedrine [6]. Altering the patient's position to a supine position can also help hemodynamics [9].

Our patient was predisposed to the BJR: (1) she had a history of vasovagal (neurocardiogenic?) syncope, (2) she possessed a thin body frame, which likely put her in a sensitive volume state, (3) she received limited intravenous fluids perioperatively to avoid hypertension, and (4) she was in a beach chair position for surgery, causing venous pooling. Hypovolemia (relative or absolute) could increase the risk of vasodepressor and bradycardic responses [2]. Likewise, short- and long-acting local anesthetic with epinephrine were utilized for the procedure.

Other etiologies of symptomatic bradycardia and hypotension in cranial neurosurgery include venous air embolism and the trigeminal cardiac reflex (TCR) (Table 1). Air embolism typically occurs during trephination, with an episode of coughing followed by a “swoon” in blood pressure [12, 13]. However, in the case presented here, the episode occurred 90 minutes after her previous burr hole was exposed. There was no new trephination, coughing episode, or elevation in respiratory rate to suggest that venous air embolism was the underlying etiology. Moreover, symptoms from venous air embolism would be gradually progressive and improve only after appropriate therapeutic maneuvers were performed. Our patient had a rapid clinical course, which was inconsistent with a venous air embolism.

The TCR involves stimulation of the trigeminal nerve or sensory branches, which leads to activation of the vagal motor nucleus and inhibition of the heart and systemic vascular system [1, 14]. Placement of a stereotactic frame can elicit the TCR if a pin was erroneously placed on the supraorbital nerve [15]. Irritation of the dura mater (i.e., cauterization of the cerebellar tentorium [16], evacuation of a subdural empyema [17], embolization of dural-based vascular pathologies [14, 18], and resection of a falcine meningioma [19]) may also elicit the reflex. Predisposing factors include the use of certain perioperative medications (beta blockers, calcium channel blockers, sufentanil, and alfentanil), a history of high vagal tone, and the presence of hypercapnea or hypoxemia [16, 20–22]. On the other hand, the depth of sedation and anesthesia may play a role in blocking the reflex [22, 23]. Although the patient was not sedated during implantation of the DBS macroelectrode, irritation of the dura was likely minimal during implantation. Table 1 summarizes the key differences between venous air embolism, TCR, and BJR.

## 4. Conclusions

With neurosurgery, intraoperative symptomatic bradycardia and hypotension can produce severe consequences if not managed appropriately. The hemodynamic instability may be explained by venous air embolism, the TCR, or the BJR. Previous instances during DBS surgery have been ascribed to venous air embolism during trephination. This report describes a precipitous episode with eventual asystole that was likely mediated by the BJR. This has not been documented previously with DBS surgery. The patient's threshold for the BJR was lowered due to her underlying history, coupled with hypovolemia (based on fluid management, body habitus, and beach chair positioning) and the use of epinephrine as a local anesthetic. After appropriate preventive interventions, the surgery was performed uneventfully.

## Figures and Tables

**Table 1:** Comparison of various etiologies of symptomatic bradycardia and hypotension.

	Venous air embolism	Trigeminal cardiac reflex	Bezold-Jarisch reflex
Incidence	Up to 4.5% in DBS surgeries [10]	Up to 18% in neurosurgery series [11]. No known prior reports in DBS surgery	No known prior reports in neurosurgery

Triggers	Trephination	Irritation of trigeminal nerve or sensory branches	Hypovolemia, spinal anesthesia leading to decrease preload

Mechanism	Entrance of air into venous system	Afferent limb, stimulation of the trigeminal nerve or sensory branches	Afferent limb, cardiac receptors via nonmyelinated type C vagal fibers
		Efferent limb, activation of vagal motor nucleus and inhibition of heart and systemic vascular system	Efferent limb, intramyocardial C fibers can potentiate a sudden withdrawal of sympathetic outflow, increasing vagal tone

Presentation	ST-T changes, right heart strain, oxygen desaturation, low end tidal CO_2_, coughing, wheezing, chest pain, “swoon,” and so forth	Bradycardia, hypotension, apnea, and gastric hypermotility	Bradycardia, hypotension

Predisposing factors	Sitting position, semisitting position	Use of medications (beta blockers, calcium channel blockers, sufentanil, and alfentanil), history of vagal episodes, presence of hypercapnea or hypoxemia, and light anesthesia	History of neurocardiogenic syncope, hypovolemia, medications (local anesthetic with epinephrine), sitting position, decreasing preload, and venous blood pooling

Treatment	Obtaining hemostasis, irrigation of surgical field, leveling patient's head to right atrium in left lateral decubitus, and use of central venous catheter for aspiration of air	Increased depth of anesthesia (i.e., propofol bolus)	Immediate fluid resuscitation, vagolytics (atropine and glycopyrrolate), ondansetron, metoprolol, and ephedrine
